# Editorial: Cerebral cavernous malformations

**DOI:** 10.3389/fneur.2025.1687562

**Published:** 2025-09-17

**Authors:** Susanna M. Zuurbier, Alejandro N. Santos, Kelly D. Flemming, Giuseppe Lanzino, Philipp Dammann, Mirjam R. Heldner

**Affiliations:** ^1^Department of Neurology, University Hospital Antwerp, Edegem, Belgium; ^2^Department of Neurosurgery, University Hospital of Essen, Essen, Germany; ^3^Department of Neurology, Mayo Clinic, Rochester, NY, United States; ^4^Department of Neurosurgery, Mayo Clinic, Rochester, NY, United States; ^5^Department of Neurology, University Hospital Bern, Bern, Switzerland

**Keywords:** stroke, cavernous, malformation, treatment, medication

Cerebral cavernous malformations (CCMs) are a rare type of cerebral vascular abnormality, characterized by variable localization and clinical presentation. Although seizures, focal neurological deficits, and intracerebral hemorrhage are the classic presentations, there is still no consensus on the optimal management of the lesions. This clinical uncertainty is compounded by limited lack of robust evidence to guide treatment decisions. While neurosurgical excision and stereotactic radiosurgery can provide symptomatic relief, they also carry risks—including postoperative deficits and radiation-induced injury. Conversely, conservative monitoring may leave patients vulnerable to recurrent hemorrhage. A more nuanced understanding of the natural history of CCMs, is urgently needed to support informed, shared decision-making.

Notably, sex-specific factors in CCM pathophysiology and management are increasingly being recognized. Hormonal influences—particularly from exogenous estrogen and progesterone—may modulate lesion behavior. As Bektas et al. observed, such hormones may increase hemorrhagic risk and impact seizure patterns, highlighting the need for tailored counseling in women of reproductive age. Individuals with CCM who are considering hormone therapy should undergo a comprehensive risk assessment. This includes evaluating their individual risk of symptomatic hemorrhage and its potential morbidity, alongside the risks, benefits, and alternatives to hormone therapy. Interestingly, pregnancy does not appear to elevate hemorrhage risk, though this remains an area where evidence is still evolving. Regarding childbirth, concerns have been raised about the potential for intracerebral hemorrhage in patients with vascular malformations during labor. However, intracerebral hemorrhage from a CCM during delivery has only rarely been documented. For patients with stable CCM, vaginal delivery is generally considered safe and appropriate. In cases involving recent neurological symptoms—such as a significant focal deficit or a recent hemorrhage—a cesarean section may be considered as an alternative.

The disease burden of CCMs extends well beyond physical symptoms. A study by Meessen et al. involving patients with familial CCMs revealed a high prevalence of depression (32%), which appeared lower in those treated with propranolol over 2 years compared to those without propranolol treatment (29% vs. 56%). Anxiety levels, measured using the State–Trait Anxiety Inventory X1 and X2 scales, remained stable over time. Quality of Life, assessed with the Short Form 36 and divided into physical and mental component scales, showed that familial CCM patients had lower scores on the physical component compared to the general Italian population, while mental component scores were similar. No significant effect of propranolol was observed on either the physical and mental component scales. Although these findings are not derived from a randomized trial, they underscore the important intersection between neurological disease and mental health—an area that warrants greater clinical attention.

Pharmacological strategies are also being revisited in light of new observational data. In a large cohort of 1,116 patients with sporadic CCMs, Chen et al. found an association suggesting a lower rate of hemorrhage at presentation among patients receiving antithrombotic therapy, challenging long-standing assumptions about contraindications. Cox regression analysis did not demonstrate a statistically significant association between medication intake and the risk of (re-)hemorrhage. Although beta blockers, statins, and thyroid hormones showed no association with hemorrhage risk, these insights pave the way for future trials evaluating medical strategies for risk modification.

The intersection between CCM and acute ischemic stroke management further complicates clinical decision-making. Case reports suggest that thrombolysis may carry an increased risk of hemorrhage in this population, although data remain sparse and potentially biased. Wang et al. have proposed endovascular treatment by balloon angioplasty as a promising alternative in carefully selected patients. However, further research is warranted to better understand the safety and efficacy of endovascular treatment in acute ischemic stroke patients with CCM, given the currently limited evidence.

The clinical and therapeutic landscape of cerebral cavernous malformations is continually evolving. Increasing awareness of sex-specific factors, mental health implications, and treatment responses—particularly to pharmacological interventions—may help pave the way for more personalized and patients-centered approaches to care.

